# Variant Ionotropic Receptors in the Malaria Vector Mosquito *Anopheles gambiae* Tuned to Amines and Carboxylic Acids

**DOI:** 10.1038/srep40297

**Published:** 2017-01-09

**Authors:** R. Jason Pitts, Stephen L. Derryberry, Zhiwei Zhang, Laurence J. Zwiebel

**Affiliations:** 1 Department of Biological Sciences, Vanderbilt University, Nashville, Tennessee, USA; 2 Vanderbilt Institute for Global Health, Nashville, Tennessee, USA; 3 College of Forestry, Shanxi Agricultural University, Shanxi, P. R. China; 4 Department of Pharmacology, Vanderbilt Brain Institute, Program in Developmental Biology and Institute of Chemical Biology, Vanderbilt University Medical Center, Nashville, Tennessee, USA

## Abstract

The principal Afrotropical human malaria vector mosquito, *Anopheles gambiae*, remains a significant threat to global health. A critical component in the transmission of malaria is the ability of *An. gambiae* females to detect and respond to human-derived chemical kairomones in their search for blood meal hosts. The basis for host odor responses resides in olfactory receptor neurons (ORNs) that express chemoreceptors encoded by large gene families, including the odorant receptors (ORs) and the variant ionotropic receptors (IRs). While ORs have been the focus of extensive investigation, functional IR complexes and the chemical compounds that activate them have not been identified in *An. gambiae*. Here we report the transcriptional profiles and functional characterization of three *An. gambiae* IR (*AgIr*) complexes that specifically respond to amines or carboxylic acids - two classes of semiochemicals that have been implicated in mediating host-seeking by adult females but are not known to activate *An. gambiae* ORs (*AgOr*s). Our results suggest that *AgIr*s play critical roles in the detection and behavioral responses to important classes of host odors that are underrepresented in the *AgOr* chemical space.

At least half the world’s population is at risk for malaria, while an even larger proportion of people are at risk for many other mosquito-borne diseases, including the arboviruses that cause dengue fever, yellow fevers and Zika^1^. Blood meal host selection by female mosquitoes is one of the most important factors impacting vector-borne disease transmission. Female mosquitoes are behaviorally activated to initiate upwind flights when stimulated by chemical cues, called kairomones, that are emitted by potential host species^2^. Some members of the *An. gambiae* s.l. Giles species complex, including *An. gambiae* s.s. (S form) and *An. coluzzii* (M form)^3^ are major sub-Saharan malaria vectors, in part because of their high propensity to acquire blood meals from human hosts^2^. Although the precise mechanisms underlying mosquito-host interactions have yet to be elucidated, many studies in the past decade have begun to unravel the molecular basis for fly and mosquito olfaction. These processes appear to be at least partly mediated by three families of chemosensory receptors that work together with accessory proteins to provide the mosquito with sensitivity and discrimination across a wide spectrum of odor space^4^,^5^. These include a superfamily of odorant receptor encoding genes (Ors), which are the best characterized chemosensory receptors to date^6^; gustatory receptors (Grs), a subset of which are known to mediate Anopheline responses to carbon dioxide^7^ and sugars^8^; and variant ionotropic receptors (Irs), which were first characterized in *Drosophila melanogaster* and have been shown to respond to amines, carboxylic acids and other environmental cues^9^,^10^,^11^,^12^,^13^,^14^,^15^. In contrast to the Or and Gr chemoreceptors that show generally high levels of sequence divergence across insect species, the Irs are more highly conserved and respond to amines and potentially other cues in *An. gambiae*^16^.

With a single exception, the family of 46 variant ionotropic receptor genes identified in *An. gambiae* (*AgIr*s) have not been functionally characterized^16^. In contrast, much is known about ionotropic receptors in *D. melanogaster* (*DmIrs*), where they are expressed in olfactory neurons of sensilla that are morphologically analogous to grooved peg sensilla found in *An. gambiae*^9^. Single sensillum recordings from these neurons illustrate a *DmIr* derived response profile that is distinct from the odor space encoded by *DmOrs*^9^,^10^,^11^,^12^,^13^. As is true for ORs, IRs functionally consist of heteromeric complexes, although unlike ORs, which all solely couple to the highly conserved Orco coreceptor, IR complexes may have one or more co-receptors that function as ligand-gated ion channels^9^,^10^,^11^,^12^,^13^,^14^,^15^. There is considerable experimental evidence that some *DmIrs* utilize *DmIr8a* as a co-receptor while others require both *DmIr25a* and *DmIr76b*, but not all three^9^,^10^,^11^,^12^,^13^,^14^,^15^. In addition to its role as a co-receptor, *DmIr25a* has recently been shown to function as a thermosensor and play a role control in establishing circadian rhythm in *D. melanogaster*^14^. Specifically, *DmIr25a* and *DmIr21a* have been shown to mediate exquisite cool temperature avoidance in *Drosophila* larvae^15^. Although most of the insect *Irs* have diverged in terms of gene numbers and coding (i.e. amino acid) sequence within and across species, the candidate co-receptors are highly conserved among insects^11^,^13^,^16^, suggesting that the assembly and function of *Irs* will be analogous across lineages. We have observed that the *AgIr*s function independently of *AgOr*s at the larval stage where *AgIr76b*, a potential *Ir* coreceptor, is required for *An. gambiae* larval responses to butylamine^16^, but virtually nothing is known about their relationships at the adult stage. This leads us to speculate that butylamine sensitivity in adult *An. gambiae* also requires the expression of *AgIr76b*, plus the coreceptor *AgIr*25a and an as yet identified “tuning” *AgIr*.

Although several of functional studies have been carried out on the *AgOr* family of chemoreceptors, very little is known about the roles of *AgIr*s in odor coding (the relationship between essential features of odor molecules and their transduction into patterns of neural activity) and function. Importantly, butylamine, lactic acid, and other carboxylic acids are activators of olfactory sensory neurons in *An. gambiae*^17^,^18^,^19^,^20^,^21^, are major components of human sweat^21^,^22^,^23^,^24^, and are known to be powerful attractants to host seeking *An. gambiae* and other mosquitoes^2^,^25^,^26^,^27^. Furthermore, functional analyses of *AgOr* sensitivities revealed a relative lack of responses to these classes of compounds, suggesting that non-*AgOr* mediated mechanisms likely underlie sensitivity to these important kairomones in *An. gambiae*^4^,^5^.

In the current study we have characterized of a subset of *AgIrs* that are expressed in the *An. coluzzii* (hereafter *An. gambiae* for consistency with gene annotations and the literature) antennae and maxillary palps^28^. We provide evidence that these *AgIrs* are expressed in subset(s) of antennal sensilla in *An. gambiae* that are distinct from *AgOr*-containing sensilla. In addition, we have characterized *AgIr* complexes comprised of specific co-receptors in the *Xenopus laevis* oocyte heterologous system that confer sensitivities to a narrow range of amines and carboxylic acids. Our results confirm that *AgIrs* function in adult peripheral chemosensory organs where they are likely to form the basis for *An. gambiae* behavioral responses to these two compound classes, which are critical for mosquito host seeking. Future studies will continue to illuminate the molecular foundations for host odor sensitivity, and specifically the roles that *AgIrs* play in this process.

## Results

### Expression of AgIr transcripts in An. gambiae head appendages

**Figure 1:**
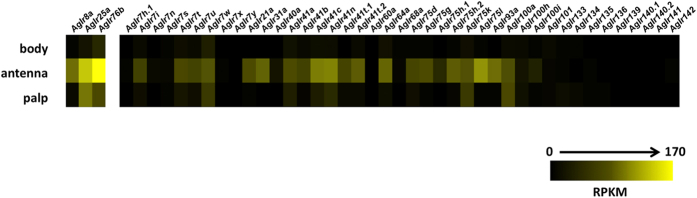
*AgIr* transcript abundances in adult appendages and larval heads. Heat map depicting abundances of *An. gambiae* variant ionotropic receptors (*AgIrs*) in adult chemosensory tissues (data adapted from^28^). Scale color intensity indicates Reads Per Kilobase per Million reads (RPKM).

We previously reported the transcript abundances of the *AgIr* family in *An. gambiae* adult antennae and maxillary palps based on RNA sequencing^28^. Here we have adapted that data set to visually display the *AgIr* transcript profile information for adult female bodies, antennae and maxillary palps (Fig. 1; Supplemental Table S1). The color intensity map in Fig. 1 indicates that the majority of *AgIr*s are expressed in the adult antennae, with considerably fewer *AgIr*s expressed in the palps. RPKM values are reported for the co-receptors, *AgIr*8a, *AgIr*25a, and *AgIr76b* as well as for the tuning receptors *AgIr41a, AgIr41c*, and *AgIr75k* that have been functionally characterized in this study (Fig. 1; Supplemental Table S1).

### Localization of AgIrs in chemosensory neurons of An. gambiae chemosensory appendages

**Figure 2:**
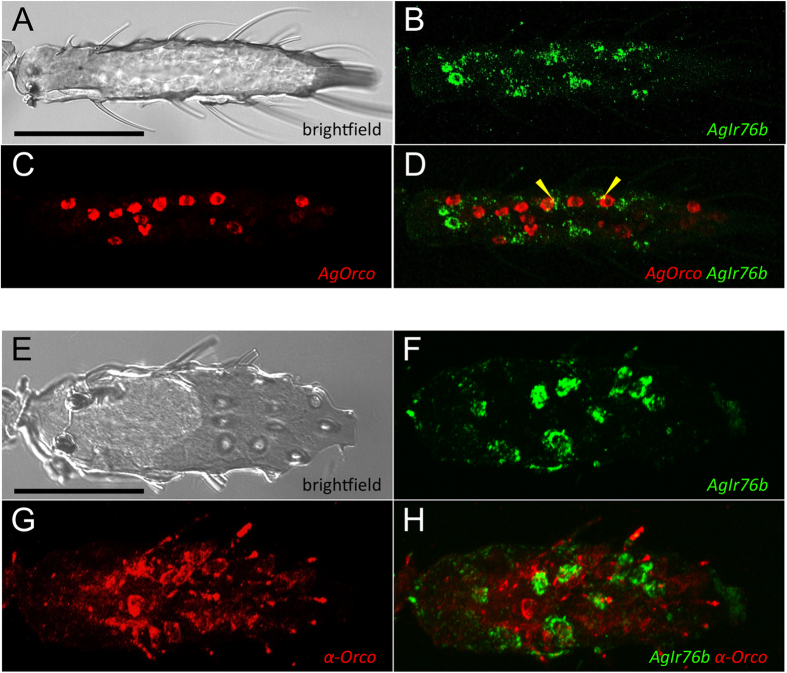
Expression of *AgIr76b* and *AgOrco* in *An. gambiae* antennae. (**A–D**) Double *in situ* hybridization using antisense RNA probes showing transcript localizations for the co-receptors *AgIr76b* (green) and *AgOr*co (red) are localized within distinct cells within adult female antennae. Yellow arrowheads indicate areas of overlap between green and red signals, due to spatial proximity of distinct cells. (**E–H**) *In situ* hybridization using an *AgIr76b* antisense RNA probe (green) plus immunohistochemical localization of AgOrco using an anti-Orco antibody (red). Scale bars ~50 μm.

Based on *DmIr* studies^9^,^10^,^11^,^12^,^13^,^14^,^15^ and our own previous observations that *AgOrco* expression is not observed in ORNs that project into grooved-peg (GP) sensilla^29^ that are activated by ammonia, a subset of carboxylic acids and other odorants^18^, we reasoned that signaling in grooved pegs is likely to be transduced by *AgIrs.* We therefore elected to further examine the relationship between *Ir* and *Orco* expression within the antennae of adult female *An. gambiae*. In order to evaluate the co-expression of *AgIr76b* and *AgOr*co in female antenna, double fluorescence *in-situ* hybridization (FISH) and single FISH, combined with immunohistochemistry (IHC) were conducted. Antennae were probed with pairs of differentially labeled *AgIr76b*- and *AgOrco*-specific riboprobes or, alternatively an anti-Orco antibody^30^. A significant number of *AgIr76b* and *AgOrco* labeled cell bodies were identified in the female antenna (Fig. 2). While we noted an apparent increase in the number of labeled neurons along the antenna from proximal to distal flagellomeres, we did not attempt to quantify those differences. This observation may indeed reflect the general apical increase in the number of olfactory sensilla in the female antennae^31^. Importantly, *AgIr76b* and *AgOrco* sense control riboprobes fail to label antennal neurons (Supplemental Figure S1) and double *AgIr76b*-*AgOrco* antisense riboprobes clearly label distinct cell bodies, as do the *AgIr76b* antisense riboprobe and anti-Orco antibody (Fig. 2E–H). Although some small puncta of apparent overlap between *AgIr76b* and *AgOrco* expression are present in the Z-stack confocal images (Fig. 2D, yellow arrowheads), we note these signals lie within subtle yet observable cellular outlines. We therefore attribute these signals to be artifacts of image stacking that are due to physical proximity or overlap of distinct cells and are not indicative of *AgIr76b* and *AgOrco* co-localization. Taken together we believe these data support the hypothesis that *AgIr76b* and *AgOrco* are not co-expressed in antennal neurons in female *An. gambiae* antennae.

While *AgIr76b*-positive neurons appeared to subtend (Fig. 2A) sensilla that do not express *AgOrco*, our studies are unable to precisely and unambiguously distinguish their exact sensillar localizations. Nevertheless, while it is possible that *AgIr76b* may be expressed in ORNs associated with other classes of sensilla, these data are consistent with a model in which *AgIr76b* is expressed in a distinctset of sensory neurons from the *AgOrco*-expressing ORNs, which we suggest should be denoted as Ionotropic Receptor Neurons (IRNs).

### Functional Characterization of AgIrs in heterologous expression assays

**Figure 3:**
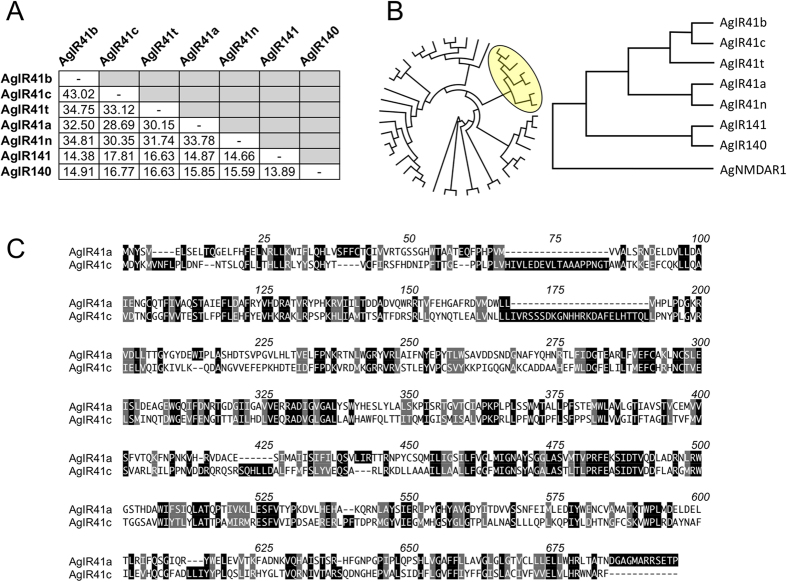
Phylogenetic relationships among AgIR41-related receptors. (**A**) Pairwise amino acid percent identity matrix. (**B**) Cladograms based on sequence alignments of all AgIRs (circular cladogram) with AgIR41 clade (yellow circle) shown using the N-Methyl-D-Aspartate Receptor 1 (AgNMDAR1) as an outgroup. (**C**) Alignment of AgIR41a and AgIR41c amino acid sequences using the single letter code. Identical residues are indicated with black boxes over white text. Similar residues are shaded with gray boxes. Numbering indicates aligned positions, including gaps.

**Figure 4:**
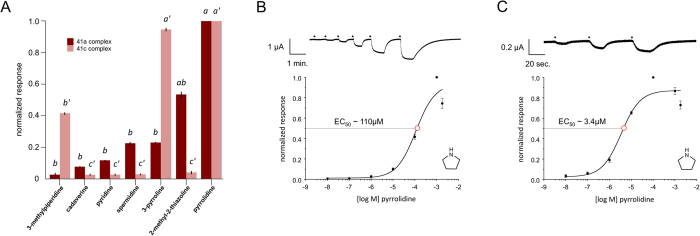
Functional analysis of AgIR41a and AgIR41c complexes in *Xenopus laevis* oocytes. (**A**) cRNAs encoding *AgIr41a* or *AgIr41c* (co-injected with *AgIr*25a and *AgIr76b*) elicit distinct profiles of chemically-evoked inward currents. Bars indicate mean normalized responses (+/− SEM) for each compound [10^−4 ^M] compared with the maximum response to pyrrolidine. Lower case letters indicate significant differences among mean responses (41a complex, *P* < 0.03 for all comparisons; 41c complex, *P* < 0.01 for a’-b’ and a’-c’ comparisons, *P* < 0.07 for b’-c’ comparisons). *n* = 4 oocytes per trial. (**B,C**) Concentration-response profiles of AgIR41a (**B**) or AgIR41c (**C**) complexes in response to pyrrolidine (closed circles) plotted on log scales. EC_50_ values (open red circles) are shown. Each data point represents the mean+/− SD (bars). Mean EC_50_ values are significantly different (*t*-test; *P* < 0.001). Representative TEVC traces showing inward current responses to increasing concentrations of pyrrolidine for each AgIR complex (top). *n* = 4 oocytes each.

In order to examine *AgIr* odor coding, we utilized oocytes to heterologously express functional OR and other chemoreceptor complexes. While the majority of *AgIr* cRNA combinations did not elicit oocyte activation against a panel of over 400 unitary odorants encompassing a broad survey of chemical classes (Supplemental Table S2), two receptors within the AgIR41a clade (Fig. 3) responded to a narrow range of compounds as described below. Coinjections of *AgIr41a* plus the candidate co-receptors *AgIr*25a and *AgIr76b* (hereafter AgIR41a complex) into oocytes conferred responses to amine/imine compounds (Fig. 4A), but not to other chemical classes in our screen. The AgIR41a complex responded to a diverse set of odorants with the nitrogenous heterocyclics 2-methyl-2-thiazoline and pyrrolidine eliciting the greatest magnitude inward currents (Fig. 4A). Control oocytes co-injected with cRNAs encoding only 2 of the 3 *AgIrs* did not respond to any of the tested compounds (Supplemental Figure S2). These data demonstrate that co-expression of *AgIrs* in oocytes is sufficient to confer sensitivity to amine/imine compounds and moreover, that *AgIr25a* and *AgIr76b* act in this instance as obligate dual co-receptors for *AgIr41a* responsiveness. Taken together, these data support the hypothesis that *AgIr*s function in a mechanistically similar manner to *DmIrs*. Importantly, *DmIr41a*, the Drosophila homolog of *AgIr41a*, confers sensitivity to 1,4 diaminobutane (putrescine) in ac2 coeloconic sensilla^13^. Moreover, *DmIr41a* and *DmIr76b* are required for behavioral attraction of fruitflies to putrescine and cadaverine^32^.

Another functional *AgIr* complex containing *AgIr41c*, a paralog of *AgIr41a* (Fig. 3B), plus the *AgIr25a/AgIr76b* coreceptors conferred sensitivity to a set of amine compounds that were distinct from those inducing responses in the AgIR41a complex (Fig. 4A). In concentration response assays, the AgIR41c complex responded with higher potency to pyrrolidine than the AgIR41a complex (Fig. 4B,C). The calculated EC_50_ values for pyrrolidine were 110 μM and 3.4 μM for the AgIR41a and AgIR41c complexes, respectively (Fig. 4B,C; Supplemental Table S3). Although AgIRs 41a and 41c are among a handful of paralogous receptors within the AgIR41 clade (Fig. 3B), they are quite divergent at the amino acid level, sharing about 29% identity and 45% similarity overall (Fig. 3C). These results indicate that divergent, yet structurally related *AgIr*s may be responsible for sensitivities to compounds within specific chemical classes. Moreover, in identifying efficacious ligand for both the AgIR41a and AgIR41c complexes, we have established positive controls for future characterization of mosquito *Irs* in the TEVC system.

**Figure 5:**
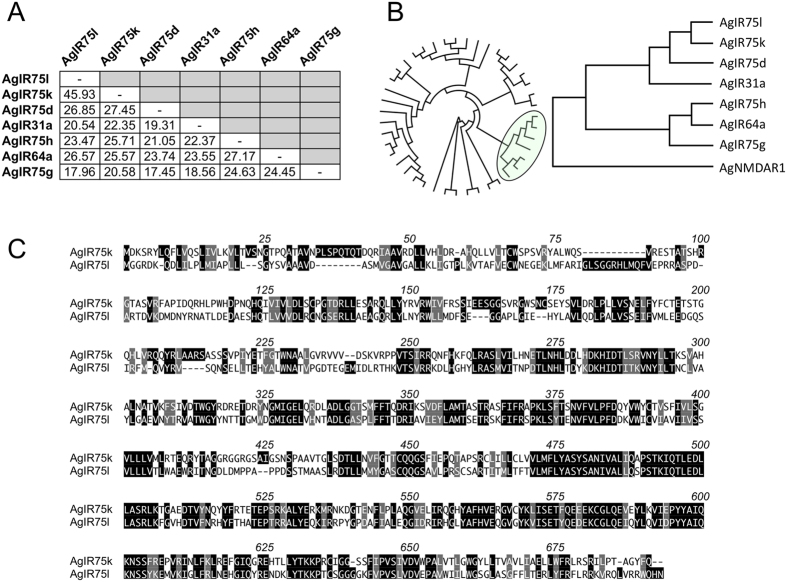
Phylogenetic relationships among AgIR75-related receptors. (**A**) Pairwise amino acid percent identity matrix. (**B**) Cladograms based on sequence alignments of all AgIRs (circular cladogram) with AgIR75 clade (green) shown using the N-Methyl-D-Aspartate Receptor 1 (AgNMDAR1) as an outgroup. (**C**) Alignment of AgIR75k and AgIR75l amino acid sequences using the single letter code. Identical residues are indicated with black boxes over white text. Similar residues are shaded with gray boxes. Numbering indicates aligned positions, including gaps.

**Figure 6:**
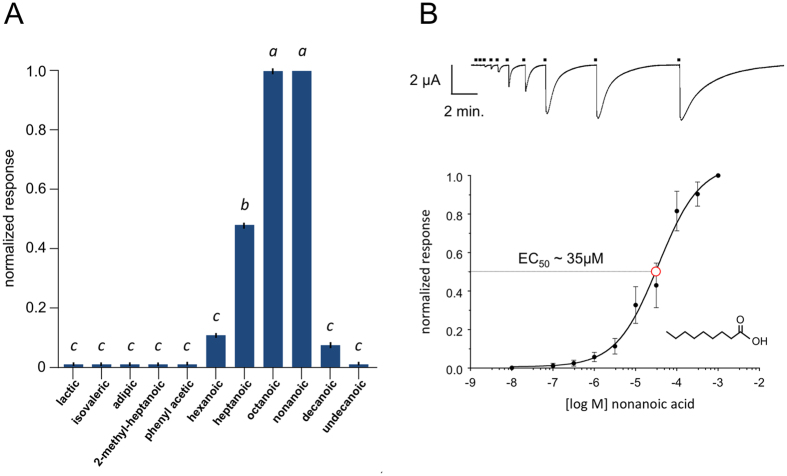
Functional analysis of the AgIR75k complex in Xenopus laevis oocytes. (**A**) cRNA encoding *AgIr75k* (co-injected with *AgIr8a*) elicits chemically-evoked inward currents in response to short chain carboxylic acids. Bars indicate mean normalized responses (+/− SEM) for each compound [10^−4^ M] compared with the maximum response to nonanoic acid. Lower case letters indicate significant differences among mean responses (*P* < 0.01 for all comparisons). *n* = 13 oocytes per trial. (**B**) Concentration-response profiles of the AgIR75k complex in response to nonanoic (closed circles) plotted on a log scale. EC_50_ value (open red circle) is shown. Each data point represents the mean+/− SD (bars). Representative TEVC trace showing inward current responses to increasing concentrations of nonanoic acid for the AgIR75k complex (top). *n* = 8 oocytes each.

In our screens we also identified a receptor from the AgIR75 clade (Fig. 5) that mediated responses to a second chemical compound class. Oocytes coinjected with cRNAs encoding *AgIr75k* and *AgIr*8a (hereafter the AgIR75k complex) responded with high inward current amplitudes to carboxylic acids (Fig. 6A). Oocytes failed to respond to the same compounds when oocytes were co-injected with cRNAs encoding *AgIr8a* plus other *AgIrs* (Supplemental Figure S3), including the closely related receptor, *AgIr75l* (Fig. 5A,C), demonstrating the specificity of the AgIR75k complex response to carboxylic acids. To further examine its functionality, oocytes expressing the AgIR75k complex were tested against a variety of carboxylic acids, among them a series of increasing carbon chain length from hexanoic (6 carbons) to undecanoic (11 carbons) acids. Of these, octanoic and nonanoic acids elicited the greatest inward currents while other structurally dissimilar carboxylic acids failed to elicit responses (Fig. 6A). A concentration response curve was generated for the AgIR75k complex in response to nonanoic acid, producing an EC_50_ in the low micromolar range (35 μM; Fig. 6B). Our observations with respect to *AgIr* responses are consistent with the hypothesis that odorant/ligand compound classes can be distinguished by their co-receptor requirements; specifically the coreceptors *Ir25a/76b* are necessary for amine sensing and the coreceptor *Ir8a* is necessary for acid sensing. This distinction has been experimentally validated in *D. melanogaster*^10^,^12^,^13^.

## Discussion

We have used RNAseq to identify the widespread expression of *AgIrs* across adult chemosensory appendages of *An. gambiae* where we hypothesize they act along with *AgOrs* and *AgGrs* to foster the detection and discrimination of semiochemicals. Interestingly, a fine-scale expression analysis employing FISH and IHC on the adult female antennae of the obligate Orco co-receptor and *AgIr76b*, one of the three potential *AgIr* co-receptors, does not support IR/OR co-localization but rather suggests cell-autonomous pathways. Indeed, the presence of multiple chemosensory receptor systems acting throughout the mosquito lifecycle, but operating via discrete networks of sensory neurons, is similar to observations made in *D. melanogaster* and other insects^13^,^33^,^34^. Either finding would be novel compared to *Ir* expression patterns in *D. melanogaster*^9^ and suggest potential functional distinctions between fruit flies and mosquitoes. Conversely, novel *AgIr* expression could indicate that these genes perform as yet uncharacterized roles as sensors of endogenous chemical cues such as biogenic amines or neuropeptides that are thought to modulate mosquito odor responses^35^,^36^,^37^,^38^.

We expected that functional *AgIr* complexes would respond to human kairomones that are known to elicit grooved peg responses, but that have not been correlated with *AgOr* sensitivity. This would further support the hypothesis that *AgIrs* underlie functionally distinct chemosensory transduction pathways that are specifically used to locate human hosts for blood meal acquisition by female *An. gambiae*. By extension, *AgIr* homologs are likely to underlie similar response pathways in other mosquito species. Such a correlation would be a significant step forward in both enhancing our understanding of the genetic basis of anthropophagy in this organism and would also identify an important new set of molecular targets for the development of novel chemical agents that could interfere with host seeking mosquito behavior, especially to host preference. It is possible that one or more such compounds will become the next generation of personal protective repellents, or surveillance trap-enhancing attractants that can be utilized in integrated vector management programs to help reduce mosquito-borne disease transmission.

In this study, we have defined and functionally characterized the first three *AgIr* complexes using the oocyte heterologous expression system and two-electrode voltage clamp electrophysiology. The saturated heterocyclic amine pyrrolidine, with a characteristic “spermous” odor^39^ is a ligand for both the AgIR41a and AgIR41c complexes eliciting robust responses, albeit with differing potencies. This apparent redundancy in sensitivity suggests that either that pyrrolidine may be combinatorially encoded *in vivo* or that structurally related compound(s) elicit more potent responses in one or both complexes. It is noteworthy that gravid *Ae. aegypti* females have recently been shown to be attracted to the polyamines putrescine and cadverine in oviposition assays^32^ supporting a potential role for IRs in mosquito oviposition site selection.

The stimuli that activate the AgIR75k complex are of particular interest as carboxylic acid-containing compounds are a major component of human sweat^22^,^23^,^24^,^25^ and are likely to be vitally important for female host seeking^40^. The AgIR75k complex may therefore provide a molecular basis for the host-seeking behavior of this mosquito species. The novel functional data for *AgIrs* reported and together with a previous study defining a role for *AgIr76b* in mediating larval responses to butylamine^16^ further supports the importance this highly conserved gene family in mosquito chemoreception. Interestingly, butylamine did not induce responses in either the AgIR41a or AgIR41c complexes, implying that another *AgIr* complex is responsible for this sensitivity in larvae.

The ongoing functional characterization of a second major family of chemoreceptors in *An. gambiae* is an important step forward in our understanding of chemosensation in an insect that continues to have a dramatically negative impact on human health. Of particular significance in this work is the identification of receptor complexes that likely underlie mosquito sensitivities to some of the most important chemical classes that are known to elicit host-seeking behavior. For example, ammonia and lactic acid act synergistically to increase the attractiveness of either odor blends or live hosts to *An. gambiae* and other vector mosquitoes^26^,^27^,^41^,^42^,^43^,^44^,^45^,^46^,^47^,^48^,^49^,^50^,^51^,^52^,^53^,^54^. It is noteworthy that *DmIrs* have been implicated in ammonia and acid sensing in *D. melanogaster*^9^,^12^,^13^,^55^. Although we may indeed find that the general principles of *Ir* function are conserved, the potential differences, subtle or pronounced, would be of interest to many different research communities including evolutionary biology, medical entomology/vector biology, and neurobiology, among others.

Molecular approaches have been successfully used to identify compounds that hyperstimulate ORNs in mosquitoes, thus providing a basis for the development of next generation repellents^56^,^57^,^58^. Indoor residual spraying (IRS) and insecticide treated bednets (ITNs) are the frontline vector control strategies adopted by many of the world’s most malaria endemic countries^59^,^60^. The potential negative impacts on human health and residual environmental hazards notwithstanding, insecticides have historically lost their effectiveness as mosquitoes have developed resistance to their toxicities through multiple mechanisms. Currently, pyrethroids are the only class of insecticides in use in many countries, exacerbating the potentially devastating effects of resistance. Thus, the development of alternative vector control methods may become critical components of integrated insect management programs, especially as outbreaks of arboviral diseases such as West Nile, Chikungunya and Zika become increasingly more widespread whilst malaria continues to burden much of the world’s population.

## Methods

### Statement of Research Ethics

All research was conducted in accordance with the NIH Guidelines for Research Involving Recombinant or Synthetic Nucleic Acid Molecules and the Vanderbilt University Environmental Health and Safety policies. Lab containment practices, equipment and facilities were reviewed and approved by the Vanderbilt Institutional Biosafety Committee (IBC).

### Mosquitoes and mosquito rearing

*An. coluzzii* (SUA 2La/2La) originating from Suakoko, Liberia, previously known as *Anopheles gambiae s.s*. M form^3^, was maintained at 27 degrees centigrade, 75% relative humidity under a 12:12 light– dark cycle in the Vanderbilt Insectary as previously described^61^. As noted in the text, while acknowledging and indeed embracing the recent change in the species nomenclature for *An. coluzzii*^3^, we have elected to nevertheless maintain the use of *An. gambiae* throughout this report in order to maintain consistency with the previously published literature as well as with pre-existing gene annotations.

### RNA sequencing and Transcript Abundances

Quantification of *AgIr* transcripts for adult female antennae and maxillary palps were derived from previously published data^28^. mRNA isolation and cDNA library preparation were carried out using the *Illumina* mRNA sequencing kit (*Illumina* Inc.; San Diego, CA). Libraries were sequenced using an *Illumina* HiSeq2000. Raw read files were quality checked, uniformly trimmed to account for spurious adapter incorporation (5′end) and for sequencing reaction degeneration (3′-end) and mapped to the *An. gambiae* genome (http://www.VectorBase.org). Mapping was carried out using seqmap software, configured to allow for a maximum of three mismatches per read. Processed mapping data was then consolidated based upon AGAP number and the results summarized by rseq software. Total weighted reads and AGAP transcript lengths were used to calculate a normalized transcript abundance level in units of Reads Per Kilobase per Million reads mapped (RPKMs)^62^.

### Amino Acid Alignments AgIr Phylogenic Reconstructions

*AgIr* amino acid sequences were aligned using MUltiple Sequence Comparison by Log-Expectation (MUSCLE^63^). Phylogenetic trees were constructed using the neighbor-joining method without distance correction^64^.

### Fluorescence *in Situ* Hybridization with Immunohistochemistry

Full length or partial *AgIr* clones were generated from cDNA prepared from female *An. gambiae* appendages via PCR-amplification (Invitrogen, Inc.) and subsequently subcloned TOPO-TA dual promoter vector (Invitrogen, Inc.). Digoxigenin (DIG) or fluorescein (FITC)-labeled fluorescence *in situ* hybridization (FISH) riboprobes were transcribed from linearized plasmids containing *AgIr* gene fragments using an RNA labelling mix (Roche Life science, IN, USA) and T7/SP6 RNA polymerase (New England Biolabs, Inc.) under the manufacture’s recommended protocols. Antisense experimental, and sense control, probes were conducted in parallel.

Single riboprobe and antibody studies were performed as described previously^65^ with the following modifications. Briefly, the antennae were dissected from 4–6 days old female heads and transferred to PBS-T solution immediately (4% paraformaldehyde in 1X RNase-free PBS, 0.1% Triton X-100). Antennae were cryosectioned at 8.0–10.0 μm and collected on Superfrost Plus slides. After fixation with 4% paraformaldehyde in 1X RNase-free PBS for 10 minutes at room temperature, the slides were washed 3 times at room temperature for 2 minutes each in 1X RNase-free PBS, acetylated for 10 minutes (0.1% triethanolamine, 1/400X volume acetic anhydride) at room temperature, and washed in 1X RNase-free PBS at room temperature. Slides were placed in prehybridization solution (50% deionized formamide, 5X SSC, 50 μg/mL heparin sodium salt, 0.1% tween-20) in a 65 °C 5X SSC humidified chamber for 1–2 hours. 40–100 ng antisense or sense riboprobes were diluted into approximately 150 μL of prehybridization solution and heated to 90 °C for 5 minutes, then directly placed on ice. The riboprobes were added to each corresponding slide and a glass coverslip was placed in order to prevent evaporation during hybridization. Slides were placed in a 65 °C 5X SSC humidified chamber for 18–24 hours. Following hybridization, each slide was washed in 5X SSC at 65 °C to facilitate removal of coverslips, followed by a triplicate series of 20-minute high-stringency washes in 0.2X SSC at 65 °C. Slides were washed in TN buffer (0.1% Tris-HCl, 0.15% NaCl) at room temperature for 10 minutes and TNB buffer [0.1% Tris-HCl, 0.15% NaCl, 0.5% Blocking reagent (Perkin Elmer, USA)] for 30–60 min at room temperature.

Following riboprobe hybridization and washing, slides were incubated with 1:500 anti-Digoxigenin-POD (Roche life science, IN, USA) and 1:500 anti-Orco antibody^30^ diluted in TNB buffer in a humidified chamber overnight at 4 °C and then washed 3 times in TNT buffer (0.1% Tris-HCl, 0.15% NaCl, 0.05% tween-20) for 5 minutes each at room temperature. Subsequently, secondary anti-rabbit cyanine-3 coupled antisera was diluted 1:500 in TNT buffer (0.1% Tris-HCl, 0.15% NaCl, 0.05% Tween-20) and applied for 1 hour at room temperature, then washed 3 times in TNT buffer for 5–10 minutes each with light agitation. Riboprobe signals were developed using tyramide signal amplification (TSA) reagents (PerkinElmer, USA) according to the manufacturer’s instructions for approximately 10 minutes at room temperature in the dark and washed 3 times in TNT buffer for 5–10 minutes each with light agitation. Slides were mounted with Vectashield (Vector Labs, USA) and visualized using either an Olympus BX-60 fluorescence microscope or a Zeiss LSM 510 META Inverted Confocal microscope.

The protocol for double FISH was unchanged with the exception of the inclusion of an additional set of antisense and sense probes to the hybridization mixtures. After riboprobe hybridization and stringency washes as detailed above, slides were incubated with 1:500 anti-Digoxigenin-Peroxidase (POD) (Roche Life science, IN, USA) diluted in TNB buffer in a humidified chamber for 1–3 hours at room temperature and then washed 3 times in TNT buffer with light agitation for 5 minutes each at room temperature. Anti-Digoxigenin-POD signal was developed using Tyramide Signal Amplification (TSA) reagents (Perkin Elmer, USA) according to the manufacturer’s instructions for approximately 10 minutes at room temperature in the dark, then washed 3 times in TNT buffer for 5–10 minutes each with light agitation. The POD enzyme was quenched by placing slides in a 3% H2O2 solution diluted in TNT for 30 minutes at room temperature. The slides were washed 3 times in TNT buffer for 5–10 minutes each with light agitation, placed in TNB for 30 minutes at room temperature and thereafter incubated with anti-fluorescein-POD diluted 1:500 in TNB buffer in a humidified chamber for 1–3 hours at room temperature followed by washing 3 times in TNT buffer with light agitation for 5 minutes each at room temperature. Anti-fluorescein-POD signal was developed using TSA reagents (Perkin Elmer, USA) according to the manufacturer’s instructions for approximately 10 minutes at room temperature in the dark, then washed 3 times in TNT buffer for 5–10 minutes each with light agitation. Slides were mounted with Vectashield and visualized using either an Olympus BX-60 fluorescence microscope or a Zeiss LSM 510 META Inverted Confocal microscope.

### cDNA Plasmid Construction and cRNA synthesis

Full-length coding sequences of *AgIr* cDNAs, based on current gene annotations^16^, were amplified by RT-PCR from adult *An. gambiae* antennae. Primers specific for the coding sequences of the *AgIrs* used were constructed using the predicted sequences as described^16^ (Supplemental Table S4). The Gateway® cloning system (Invitrogen, Inc.) was used for all *AgIr* cloning and plasmids were verified by sequencing prior to subcloning into the destination vector, pSP64T, for *in vitro* transcription and heterologous expression in Xenopus oocytes. *In vitro* synthesis of 5′ capped complementary RNAs (cRNAs) for *AgIr* constructs were carried out using the mMESSAGE mMACHINE *in vitro* expression kits according to the manufacturer’s protocol (Ambion, Inc.).

### Xenopus laevis Oocyte Retrieval, Microinjection, and Incubation

All *Xenopus laevis* protocols were carried out with the approval of the Vanderbilt University Institutional Animal Care and Use Committee. Expression and functional characterization of *AgIr* complexes were conducted essentially as described for *AgOr* proteins^4^. *X. laevis* ovarian lobes were extracted from fully mature females via laparotomy. An incision less than 1 cm was made ventrally, lateral of the body midline, and along the ovarian wall. Ovarian lobes were extracted and placed in a washing buffer (96 mM NaCl, 2 mM KCl, 5 mM MgCl2, and 5 mM HEPES, pH 7.6) supplemented with 0.1% gentamycin. The ovarian lobes were gently separated via forceps and subsequently treated with 2 mg/mL collagenase S-1 in washing buffer for 1.5 hours at room temperature while on a laboratory shaker. Individual oocytes were incubated in ND96 buffer (96 mM NaCl, 2 mM KCl, 1 mM MgCl2, 1 mM CaCl2, and 5 mM HEPES, pH 7.5) supplemented with 5% dialyzed horse serum, 50 μg/mL tetracycline, 100 μg/mL streptomycin, and 550 μg/mL sodium pyruvate at 18 °C until microinjection. Each oocyte was injected with 27.6 nL of an *AgIr* cRNA mixture containing a tuning *AgIr* and one or more potential co-receptor subunits (i.e. *AgIr*s 25a + 76b or *AgIr*8a). After injection, oocytes were incubated separately at 18 °C in supplemented ND96 buffer. Oocytes were allowed to incubate for 3 to 5 days until assayed by two-electrode voltage clamp electrophysiology.

### Two-Electrode Voltage Clamp (TEVC) Electrophysiology

Whole-cell currents were recorded from injected oocytes using the OC-725C feedback amplifier (Warner Instruments). This amplifier was connected to a computer interface through the Axon Digidata 1440 A Digitizer; data acquisition and analysis were carried out using pCLAMP 10 software (Axon Instruments). The current-injecting and voltage-sensing electrodes were made of a Ag/AgCl wire housed in a pulled borosilicate glass capillary containing 3 M KCl. Each oocyte was clamped at a holding potential of −80 mV while being perfused with buffer.

### Chemical Stimuli

Approximately 400 unitary compounds of the highest commercial grade were tested in this study (Table S1). One molar (1 M) stocks of each compounds were prepared in DMSO and subsequently diluted in ND96. For initial compound screens, mixtures of 10–12 compounds of the same functional class were prepared at 1 × 10^−4 ^M each and pipetted in the TEVC perfusion system. Compounds from mixtures that elicited oocyte responses were tested individually to identify ligand-receptor pairings. A minimum threshold of 50 nA was used as a cutoff for positive responses to screened compounds, roughly equal to the noise level in the system. For the most efficacious compounds, concentration response profiles were generated using multiple oocytes.

### Statistical Treatment of Data

Descriptive statistics, including arithmetic means, standard deviations and standard errors of the mean were calculated using *Microsoft* Excel. The R statistical package (www.r-project.org) was used for subsequent analyses, except where otherwise indicated. Oocyte inward currents were normalized by dividing individual responses by the maximum response per trial, followed by calculating the arithmetic mean of normalized responses for each compound. Assumptions of normality and equal variance were confirmed with the Shapiro-Wilk and *F*-tests, respectively, and arithmetic means for each treatment in the oocyte TEVC functional assays were analyzed for statistical significance by single-factor ANOVA; significant results were followed up with Tukey-Kramer post-tests to distinguish among group means. Concentration response curves and EC_50_ values were calculated using Prism 4 (GraphPad Software, Inc.). The Student’s *t*-test was applied to mean EC_50_ values for *AgIr41a* and *AgIr41c* pyrrolidine responses to evaluate statistical significance.

## Additional Information

**How to cite this article**: Pitts, R. J. *et al*. Variant Ionotropic Receptors in the Malaria Vector Mosquito *Anopheles gambiae* Tuned to Amines and Carboxylic Acids. *Sci. Rep.* **7**, 40297; doi: 10.1038/srep40297 (2017).

**Publisher's note:** Springer Nature remains neutral with regard to jurisdictional claims in published maps and institutional affiliations.

## Supplementary Material

Supplemental Data

Supplementary Table S1

Supplementary Table S2

Supplementary Table S3

Supplementary Table S4
