# Supplementary material for: Variant Ionotropic Receptors in the Malaria Vector Mosquito Anopheles gambiae Tuned to Amines and Carboxylic Acids
Source: Sci Rep. 2017 Jan 9;7:40297. doi: 10.1038/srep40297 (PMC5220300; doi:10.1038/srep40297)
Supplement: Supplemental Data [file srep40297-s1.pdf]

Variant Ionotropic Receptors in the Malaria Vector Mosquito *Anopheles gambiae* Tuned to Amines and Carboxylic Acids.

R. Jason Pitts[1,2*], Stephen L. Derryberry, Jr.[1*], Zhiwei Zhang[1,3] and Laurence J Zwiebel[1,2,4]

Author Affiliations:

[1]Department of Biological Sciences, Vanderbilt University, Nashville, Tennessee, USA

[2]Vanderbilt Institute for Global Health, Nashville, Tennessee, USA

[3]College of Forestry, Shanxi Agricultural University, Shanxi, P. R. China

[4]Department of Pharmacology, Vanderbilt Brain Institute, Program in Developmental Biology and Institute of Chemical Biology, Vanderbilt University Medical Center, Nashville, Tennessee, USA

Correspondence to be sent to: Laurence J. Zwiebel, Department of Biological Sciences, Vanderbilt University, Nashville, USA. email: l.zwiebel@vanderbilt.edu

* these authors contributed equally to this work

Supplemental Figure S1. *In situ* and immunohistochemistry localization controls.

(A-D) Double *in situ* hybridization using sense RNA probes *AgIr76b* (green) and *AgOrco* (red). (E-H) *In situ* hybridization control using an *AgIr76b* sense RNA probe (green) plus immunohistochemical localization of *AgOr*co using an anti-Orco antibody (red). Scale bars ~50 µm.

Supplemental Figure S2. Control responses of *Xenopus laevis* oocytes to amine/imines.

Responses to unitary compounds [10^{-4} M] from oocytes that were co-injected with cRNAs encoding the subunits indicated in the color keys. Mean responses (+/-SEM) were normalized against the maximum responses to pyrrolidine of *AgIr41a* + *AgIr25a/76b* (A), or *AgIr41c* + *AgIr25a/76b* (B). *n* = 4 oocytes each.

Supplemental Figure S3. Control responses of *Xenopus laevis* oocytes to carboxylic acids.

Mean responses (+/-SEM) to blends of compounds (each at [10^{-4} M]) from oocytes that were coinjected with cRNAs encoding the subunits indicated in the color keys. Responses were normalized against the maximum responses to carboxylic acid blend #15 (see Table S1) of *AgIr75k* + AgIr8a. *AgIr75l* + *AgIr8a* responded with very low amplitude (~50 nA) to blend #15 in a single trial, while no other *AgIr* combinations responded to either blend. *n* = 3-5 oocytes each.

Supplemental Table S1. *AgIr* transcript abundances in *An. gambiae* chemosensory tissues. Units are given in Reads per Kilobase per Million Reads (RPKM).

Supplemental Table S2. Compounds used for screening *AgIr* responses in *Xenopus laevis* oocytes and arranged by chemical class (column A), compound name (column B) and blend groupings used in the initial screens (column C).

Supplemental Table S3. Effective concentrations at half maximal compound responses (EC_{50}) for listed *AgIr* complexes expressed in *Xenopus laevis* oocytes.

Supplemental Table S4. Cloning primers and peptide translations for full-length *AgIr* transcripts functionally characterized in this study.

Fig. S1

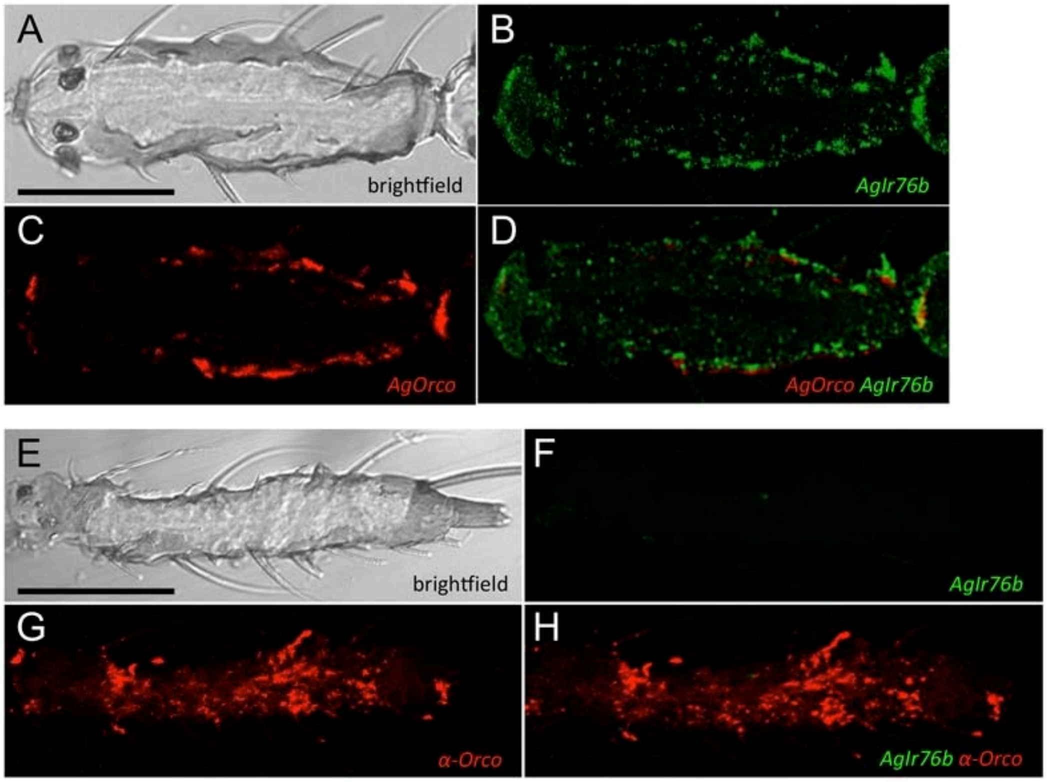
A
brightfield
B
AgIr76b
C
AgOrco
D
AgOrco AgIr76b
E
brightfield
F
AgIr76b
G
α-Orco
H
AgIr76b α-Orco

Fig. S2

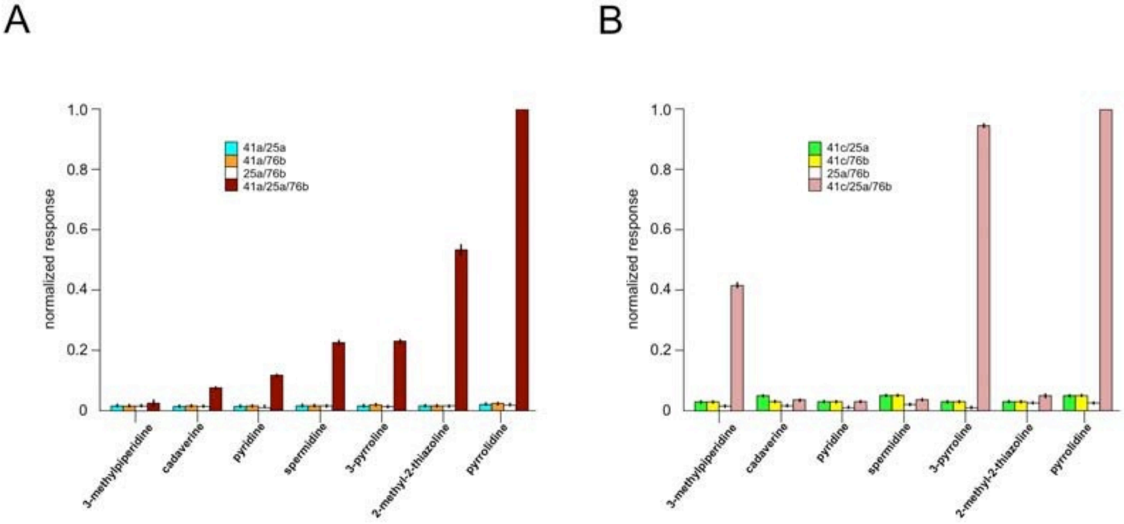
A
normalized response
1.0
0.8
0.6
0.4
0.2
0
41a/25a
41a/76b
25a/76b
41a/25a/76b
3-methylpiperidine
cadaverine
pyridine
spermidine
3-pyrroline
2-methyl-2-thiazoline
pyrrolidine
B
normalized response
41c/25a
41c/76b
25a/76b
41c/25a/76b
3-methylpiperidine
cadaverine
pyridine
spermidine
3-pyrroline
2-methyl-2-thiazoline
pyrrolidine

Fig. S3

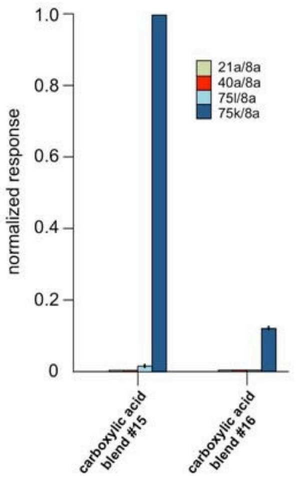
normalized response
1.0
0.8
0.6
0.4
0.2
0
21a/8a
40a/8a
75l/8a
75k/8a
carboxylic acid blend #15
carboxylic acid blend #16
